# Efficacy and safety of methylprednisolone in the prevention of seroma formation after mastectomy: Systematic review and meta-analysis

**DOI:** 10.1097/MD.0000000000045353

**Published:** 2025-10-24

**Authors:** Xiangzun Xiong, Xue Ou, Lei Wang, Xiaoyuan Zheng

**Affiliations:** a Pharmacy Department, Chongging Emergency Medical Center, Chongging, China; b Pharmacy Department, Chongging University Central Hospital, Chongqing, China; c Medical College, Chongging University, Chongqing, China; d Medical Affairs Department, The First Affiliated Hospital of Chongqing Medical University, Chongging, China.

**Keywords:** mastectomy, meta-analysis, methylprednisolone, seroma

## Abstract

**Background:**

Seroma, a common post-mastectomy complication linked to surgical inflammation, may be mitigated by anti-inflammatory agents such as methylprednisolone.

**Methods:**

This meta-analysis evaluates methylprednisolone’s efficacy and safety in preventing seroma. We systematically searched PubMed, Web of Science, Embase, Cochrane Library, China National Knowledge Infrastructure, and other Chinese databases for randomized controlled trials (RCTs) (from inception to March 2025) involving methylprednisolone use post-mastectomy. Two reviewers independently screened studies, extracted data, and assessed bias using ROB 2.0 and GRADE. Outcomes included seroma incidence, drainage metrics, wound complications, and seroma grading. Data were analyzed via Review Manager and Trial Sequential Analysis.

**Results:**

The analysis included 7 randomized controlled trials with 589 patients, among whom 294 were administered methylprednisolone. The study found that the incidence of seroma (risk ratios = 0.73, 95% confidence interval [CI] 0.54–0.98, *P* = .04), total drainage volume (mean difference  = −184.19, 95% CI: −215.30 to −153.09, *P* < .00001), and duration of drainage (mean difference  = −3.37, 95% CI: −3.98, to −2.75, *P* < .00001) were significantly lower in the methylprednisolone group compared to the control group. Remarkably, this effect didn’t extend to the incidence of wound complications (risk ratios = 0.93, 95% CI: 0.50–1.73, *P* = .82), nor did it influence seroma grading. The Trial Sequential Analysis results indicated that the evidence was sufficient and conclusive regarding the incidence of seroma, total drainage volume, and duration of drainage.

**Conclusion:**

Methylprednisolone may reduce the risk of seroma formation in patients undergoing mastectomy, along with reducing the total volume and duration of drainage. Further well-designed randomized controlled trials are needed to assess the impact of methylprednisolone on postoperative wound complications and seroma grading.

## 1. Introduction

Seroma formation is a common problem that often occurs after mastectomy and axillary dissection in patients with breast cancer, with reported incidence rates ranging from 18% to 59% in previous studies.^[1–3]^ Seroma can lead to complications such as wound infection, flap necrosis, upper limb lymphedema, and constrained limb mobility. These complications may prolong patients’ length of stay and outpatient follow-up, cause delay in subsequent adjuvant therapies, increase medical burden, and reduce patients’ quality of life.^[4–6]^

Various theories illustrate the formation of seroma, each proposing different explanations and suggesting different therapies consequently. Inflammatory response triggered by surgery is the most convincing theory among these.^[7]^ Steroid administration has been used to manage this inflammatory response in various types of surgeries, including major abdominal surgery, colectomy, head and neck surgery, plastic surgery, and cardiac surgery.^[8]^ Cyclooxygenase 2 inhibitors and steroids may reduce the synthesis of prostaglandins and leukotrienes, potentially preventing seroma formation.^[9]^

Several studies have investigated the effect of methylprednisolone on seroma formation post-mastectomy. However, the conclusions are inconsistent. For instance, in one study, following modified radical mastectomy, 46% of women developed seroma in the methylprednisolone group, compared with 78% in the saline group (*P* < .001).^[10]^ In another study, there was a slight increase in seroma formation within the methylprednisolone group; however, the differences were not statistically significant in the number of seroma aspirations after surgery between the methylprednisolone and control groups.^[7]^

In summary, there is no comprehensive evidence of the efficacy of methylprednisolone in preventing seroma formation after mastectomy, and its safety remains to be explored. This study aims to conduct a systematic review of the clinical efficacy and safety of methylprednisolone in the prevention of seroma formation after mastectomy and to provide up-to-date evidence for its clinical application and decision-making.

## 2. Methods

This systematic review and meta-analysis were conducted following the Cochrane handbook and Preferred Reporting Items for Systematic Reviews and Meta-Analyses checklist. The study was registered at the Centre for Reviews and Dissemination, University of York (PROSPERO; http://www.crd.york.ac.uk/PROSPERO, registration number CRD42023488071).

### 2.1. Literature search strategy

Relevant articles were retrieved from various databases including PubMed, Web of Science, Embase, Cochrane Library, China National Knowledge Infrastructure, Wanfang Data, Chinese Biomedicine Database and China Science and Technology Journal Database (VIP) from databases establishment to March 31, 2025. The search was conducted using the keywords “methylprednisolone,” “steroids,” “seroma,” and “mastectomy.” No date or language limitations were imposed on the search. Consultation with other relevant principal investigators and experts was done when necessary. The detailed search strategies are presented in Table S1, Supplemental Digital Content, https://links.lww.com/MD/Q410.

### 2.2. Eligibility criteria

The inclusion criteria for this study consisted of the following:

- Participants: Female patients who have undergone mastectomy or modified radical mastectomy (MRM) for breast cancer.
- Types of studies: Randomized controlled trials (RCTs).
- Intervention: patients were administered with methylprednisolone.
- Comparison: patients did not receive methylprednisolone.
- Outcomes: Incidence of seroma, total drainage volume, duration of drainage, wound complications, and seroma grading.

The exclusion criteria for this study encompassed the following: Review articles, animal studies, retrospective studies, case reports, letters to the editors, nonrandomized trials, and unavailability of full text.

### 2.3. Data extraction

Two researchers conducted a comprehensive literature review, employing established criteria to extract and cross-verify data, and any discrepancies were resolved through discussion or with the assistance of a third party. The screening process involved an initial evaluation of the paper’s title to eliminate irrelevant material, followed by a thorough examination of the abstract and full text to determine the final selection for further analysis. Data obtained consisted of the following:

- Publication date, first author and other basic information of included studies.
- Basic patient information: Type of surgery, age, BMI, administration method, etc.
- Sample size and intervention measures.
- Outcomes.
- Literature quality evaluation information.

### 2.4. Quality assessment

Two investigators independently evaluated the risk of bias for the included studies, and the findings were verified through cross-checking. We used the following updated version of ROB 2.0 assessment tools recommended by the Cochrane Collaboration,^[11]^ which collectively encompasses 5 domains: randomization process, deviation from intended interventions, missing outcome data, measurement of the outcome and selection of the reported result. For each study, the items were classified as high risk, low risk or medium (some concern) risk.

### 2.5. Statistical analysis

The feasibility of statistical analysis depended on the included studies, and data were extracted. If statistical analysis could not be conducted, only a systematic evaluation was conducted; if statistical analysis was conducted, Review Manager (version 5.4) was used for data processing. Risk ratios (RR) with a 95% confidence interval (CI) were calculated for categorical variables, and a meta-analysis was performed. The mean difference (MD) and 95% CI for continuous variables were obtained using the inverse variance method. Heterogeneity was assessed using *I*^2^: defined as high heterogeneity for *I*^2^ ≥ 75%, moderate heterogeneity for 50%≤*I*^2^ < 75%, and low heterogeneity for 25%≤*I*^2^ < 50%. A fixed-effects model was used for combining data when *I*^2^ = 0, and a random-effects model was used when *I*^2^ ≠ 0. Sensitivity or subgroup analysis was conducted to valid the robustness of results. Additionally, funnel plots were conducted to assess the presence of small study effects or publication bias in the intervention measures when the number of included studies exceeded ten.

### 2.6. Trial Sequential Analysis (TSA)

The TSA Software (0.9.5.10 Beta) was used to conduct TSA, which calculated the required information size (RIS) and evaluated the quality of the available data and conclusions derived from the meta-analysis.^[12]^ TSA boundaries were applied to prevent false-positive (type-I error) and false-negative (type-II error) results. We maintained the 2-sided type-I error rate at 5% (alpha boundary) and calculated RIS with 80% power.

### 2.7. Evaluation the strength of evidence

The strength of evidence was evaluated using the Grading of Recommendations Assessment, Development, and Evaluation (GRADE) approach. The GRADE Pro online website tool was used to create the GRADE Evidence and Summary of Findings (SoF) table,^[13]^ which indicates the overall quality of evidence for each outcome as “high,” “moderate,” “low,” or “very low.”

### 2.8. Ethics and dissemination

Ethical approval was not required because this study only involved published data.

## 3. Results

### 3.1. Studies included

**Figure 1.**
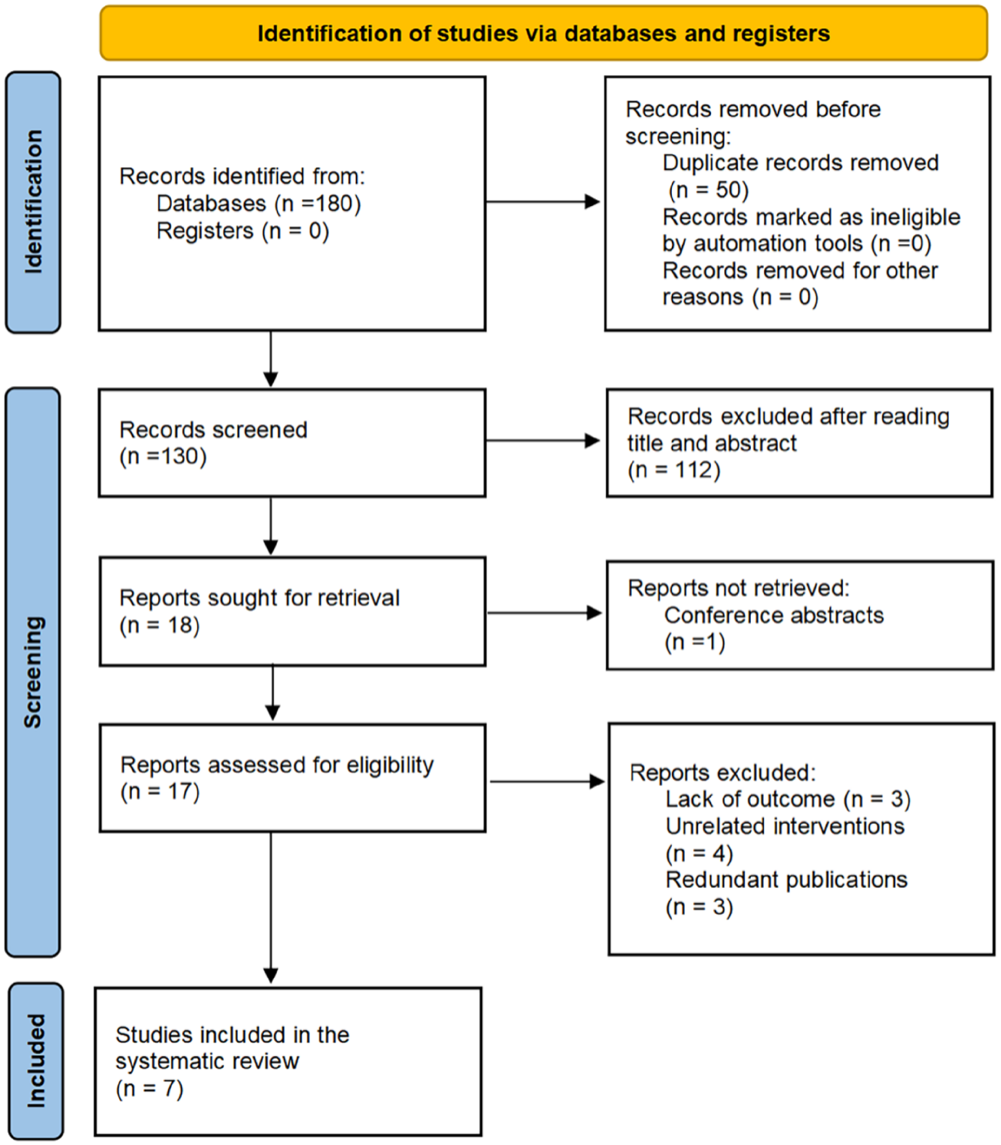
Literature search and selection.

Using the aforementioned strategy, a total of 180 articles were identified as potentially relevant. Figure 1 illustrates the literature retrieval and screening processes. The systematic review and meta-analysis included 7 RCTs that enrolled 589 patients and met all inclusion criteria.

### 3.2. Basic characteristics of included studies and the results of quality assessment

**Figure 2.**
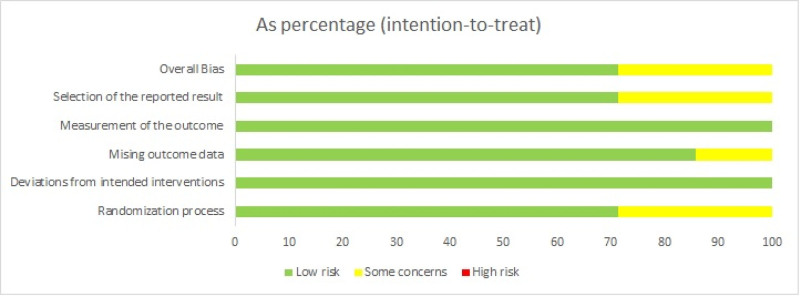
Scale map of risk assessment.

**Figure 3.**
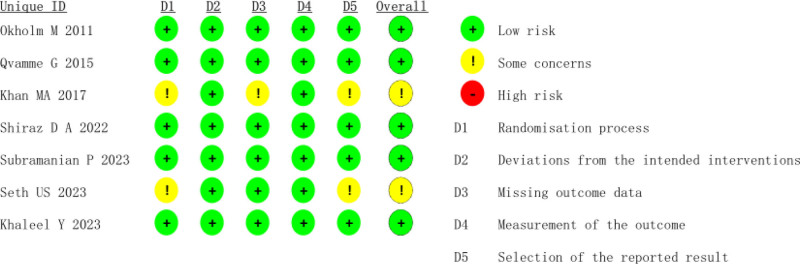
Summary map of risk assessment.

All of the studies included in this analysis were RCTs published between 2011 and 2023. The quality evaluation of each study is presented in Figures 2 and 3.

The characteristics of the included trials was listed in Table 1. The studies were conducted in Denmark (2 studies), India (2 studies), and Pakistan (3 studies), with a total of 589 patients enrolled across all 7 studies. Among them, 294 received methylprednisolone via preoperative intravenous injection or postoperative injection. The follow-up period was 10 to 30 days after surgery.

**Table 1:** Summary of detailed information about included studies.

Study	Country	Type of surgery	No. of patients(T/C)	Mean Age (T/C)	Mean BMI (T/C)	Administration method	Intervention measures		Outcomes:	Follow-up time
							T	C		
Qvamme 2015^[7]^	Denmark	M + SLNB or M + ALND or Other breast surgery	106/106	63 (61–66)/65 (62–67)	24 (24–25)/26 (24–27)	Locally injected on the first day after the operation	80 mg methylprednisolone	saline	①	30 d
Okholm 2011^[10]^	Denmark	M + SLNB or M + ALND	20/22	63.2 (48–86)/62.3 (43–79)	23.9 (16.0–36.0)/24.0 (20.4–31.1)	Intravenous injection 1.5 h before the operation	125 mg methylprednisolone	-	①②⑤	14 d
Khan 2017^[14]^	Pakistan	M or MRM or MRM + AD	33/32	32.3 ± 9.1/34.2 ± 10.1	-	Intravenous injection 1 h before the operation	120 mg methylprednisolone	-	①②③④⑤	10 d
Shiraz 2022^[15]^	Pakistan	MRM	38/38	38 ± 12.72/40 ± 11.83	-	Locally injected on the first day after the operation	80 mg methylprednisolone	saline	①	14 d
Seth 2023^[16]^	Pakistan	MRM + ALND	30/30	40.1 ± 8.1/42.1 ± 8.2	-	Intravenous injection 0.5 h before the operation	120 mg methylprednisolone	-	①②③④⑤	-
Khaleel 2023^[17]^	India	MRM	31/31	/	23.6/24.1	Intravenous injection before induction	125 mg methylprednisolone	-	①②	10 d
Subramanian 2023^[18]^	India	M + ALND	36/36	52.47 (40–70)/53.75 (38–81)	25.2/24.6	Locally injected on the first day after the operation	80 mg methylprednisolone	saline	②③⑤	30 d

AD = axillary dissection, ALND = axillary lymph node dissection, M = mastectomy, MRM = modified radical mastectomy, SLNB = sentinel lymph node biopsy; ①: incidence of seroma; ②: total drainage volume; ③: duration of drainage; ④: seroma grading; ⑤: wound complications.

### 3.3. Incidence of seroma

**Figure 4.**
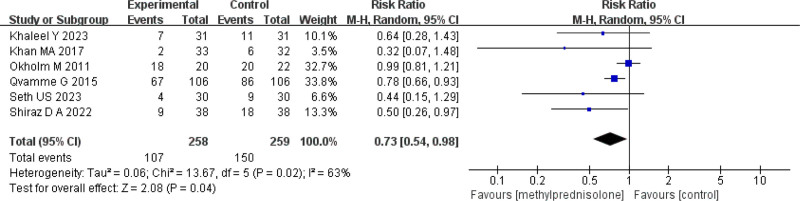
Forest plot of the incidence of seroma.

**Figure 5.**
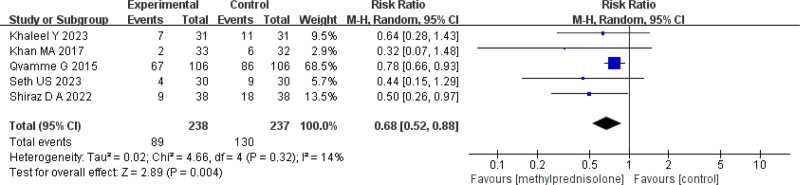
Sensitivity analysis for the incidence of seroma.

**Figure 6.**
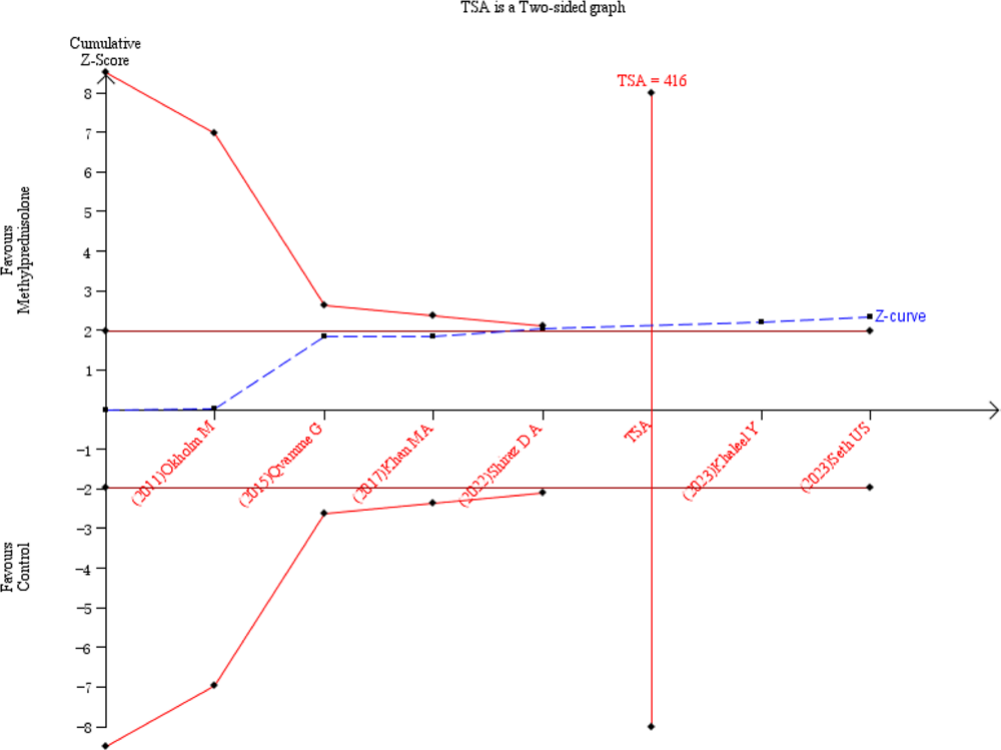
TSA for the incidence of seroma. TSA = Trial Sequential Analysis.

Six studies^[7,10,14–17]^ reported the incidence of seroma formation. There was moderate statistical heterogeneity among the studies (*P* = .02, *I*^2^ = 63%) (Fig. 4). A random-effects model was used for meta-analysis. The meta-analysis found that the incidence of seroma was significantly lower in the methylprednisolone group compared to the control group (RR = 0.73, 95% CI 0.54–0.98, *P* = .04) (Fig. 4). Sensitivity analysis showed that heterogeneity significantly decreased after excluding study^[10]^ (*P* = .32, *I*^2^ = 14%) (Fig. 5). A random-effects model was used for meta-analysis. The study found that the incidence of seroma was significantly lower in the methylprednisolone group compared to the control group (RR = 0.68, 95% CI 0.52–0.88, *P* = .004) (Fig. 5). Subgroup analysis based on different administration regimens showed that for intravenous injection before surgery, the incidence of seroma was significantly lower in the methylprednisolone group versus the control group (RR = 0.50, 95% CI 0.27–0.90, *P* = .02). However, the difference wasn’t statistically significant in the incidence of seroma between the 2 groups for local injection postoperatively (RR = 0.70, 95% CI 0.47–1.04), *P* = .08), which is shown in Table 2. The TSA demonstrated that the cumulative information size (n = 517) was 124.27% of the RIS (n = 416). The cumulative z-curve crossed the trial sequential monitoring boundary, indicating that the current evidence was sufficient and conclusive (Fig. 6).

**Table 2:** Subgroup analysis of the incidence of seroma.

Administration method	Number of included studies	Heterogeneity		Model	Statistical results		
		*P*	*I*^2^, %		RR	95% CI	*P*
Local injection after surgery	2	.18	44	Random- effects model	0.70	0.47–1.04)	.08
Intravenous injection before surgery	3	.70	0	Fixed-effects model	0.50	0.27–0.90	.02

### 3.4. Total drainage volume

**Figure 7.**
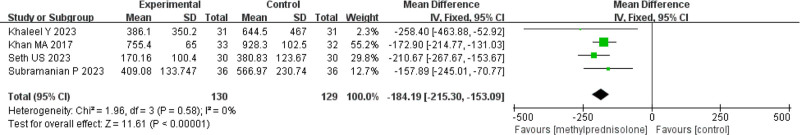
Forest plot of total drainage volume.

**Figure 8.**
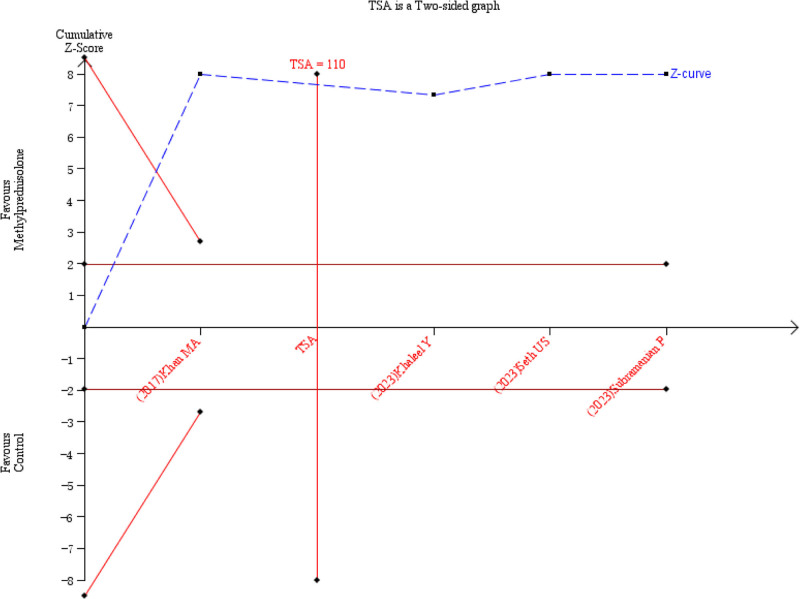
TSA for total drainage volume. TSA = Trial Sequential Analysis.

Five studies^[10,14,16–18]^ reported the total drainage volume. However, the data from one study^[10]^ could not be aggregated due to different forms of reporting. The remaining 4 studies^[14,16–18]^ showed no heterogeneity (*P* = .58, *I*^2^ = 0%) (Fig. 7). Using a fixed-effects model for meta-analysis, the results indicated that the total drainage volume in the methylprednisolone group was significantly lower than that in the control group (MD = −184.19, 95% CI −215.30 to −153.09, *P* < .00001] (Fig. 7). Subgroup analysis based on different administration regimens showed that the total drainage volume in the methylprednisolone group was significantly lower than that in the control group for both intravenous injection before surgery (MD = −188.03, 95% CI −221.33 to −154.74, *P* < .00001) and local injection after surgery (MD = −157.89, 95% CI −245.01 to −70.77, *P* = .0004] (refer to Table 3). The TSA indicated that the cumulative information size (n = 259) was 235.45% of the RIS (n = 110). The trial sequential monitoring boundary crossed by the cumulative z-curve, indicating that the current evidence was sufficient and conclusive (Fig. 8).

**Table 3:** Subgroup analysis of total drainage volume.

Administration method	Number of included studies	Heterogeneity		Model	Statistical results		
		*P*	*I*^2^, %		RR	95% CI	*P*
Local injection after surgery	1	-	-	Fixed-effects model	−157.89	−245.01 to −70.77)	.0004
Intravenous injection before surgery	3	.46	0	Random-effects model	−188.03	−221.33 to −154.74)	<.00001

### 3.5. Duration of drainage

**Figure 9.**
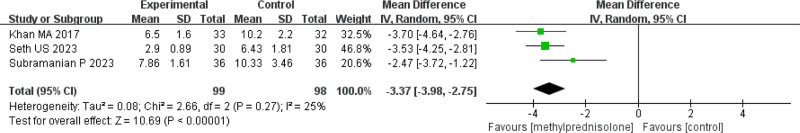
Forest plot of the duration of drainage.

**Figure 10.**
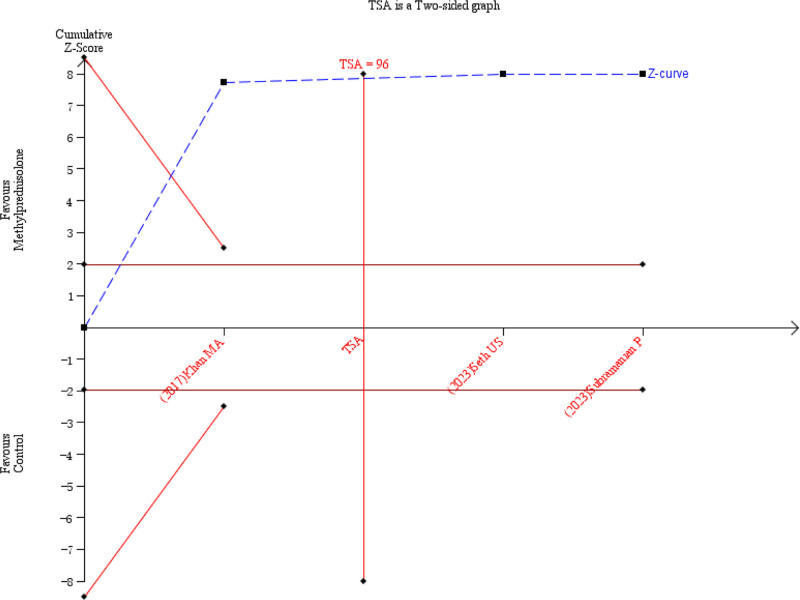
TSA for the duration of drainage. TSA = Trial Sequential Analysis.

Three studies^[14,16,18]^ reported the duration of drainage and there was low heterogeneity between studies (*P* = .27, *I*^2^ = 25%) (Fig. 9). Using a random-effects model for meta-analysis, the results showed that the duration of drainage was significantly shorter in the methylprednisolone group than in the control group (MD = −3.37, 95% CI −3.98 to −2.75, *P* < .00001) (Fig. 9). Subgroup analysis based on different administration regimens showed that the duration of drainage in the methylprednisolone group was significantly lower than that in the control group for both preoperative intravenous injection (MD = −3.59, 95% CI −4.17 to −3.02, *P* < .00001) and postoperative local injection (MD = −2.47, 95% CI −3.72 to −1.22, *P* = .0001) (Table 4). The TSA showed that the cumulative information size (n = 197) was 205.21% of the RIS (n = 96). The cumulative z-curve crossed the boundary of sequential monitoring of the study, indicating that the current evidence was sufficient and conclusive (Fig. 10).

**Table 4:** Subgroup analysis of duration of drainage.

Administration method	Number of included studies	Heterogeneity		Model	Statistical results		
		*P*	*I*^2^, %		RR	95% CI	*P*
Local injection after surgery	1	-	-	Fixed-effects model	−2.47	(-3.72, −1.22)	.0001
Intravenous injection before surgery	2	.78	0	Fixed-effects model	−3.59	(-4.17, −3.02)	<.00001

### 3.6. Wound complications

**Figure 11.**
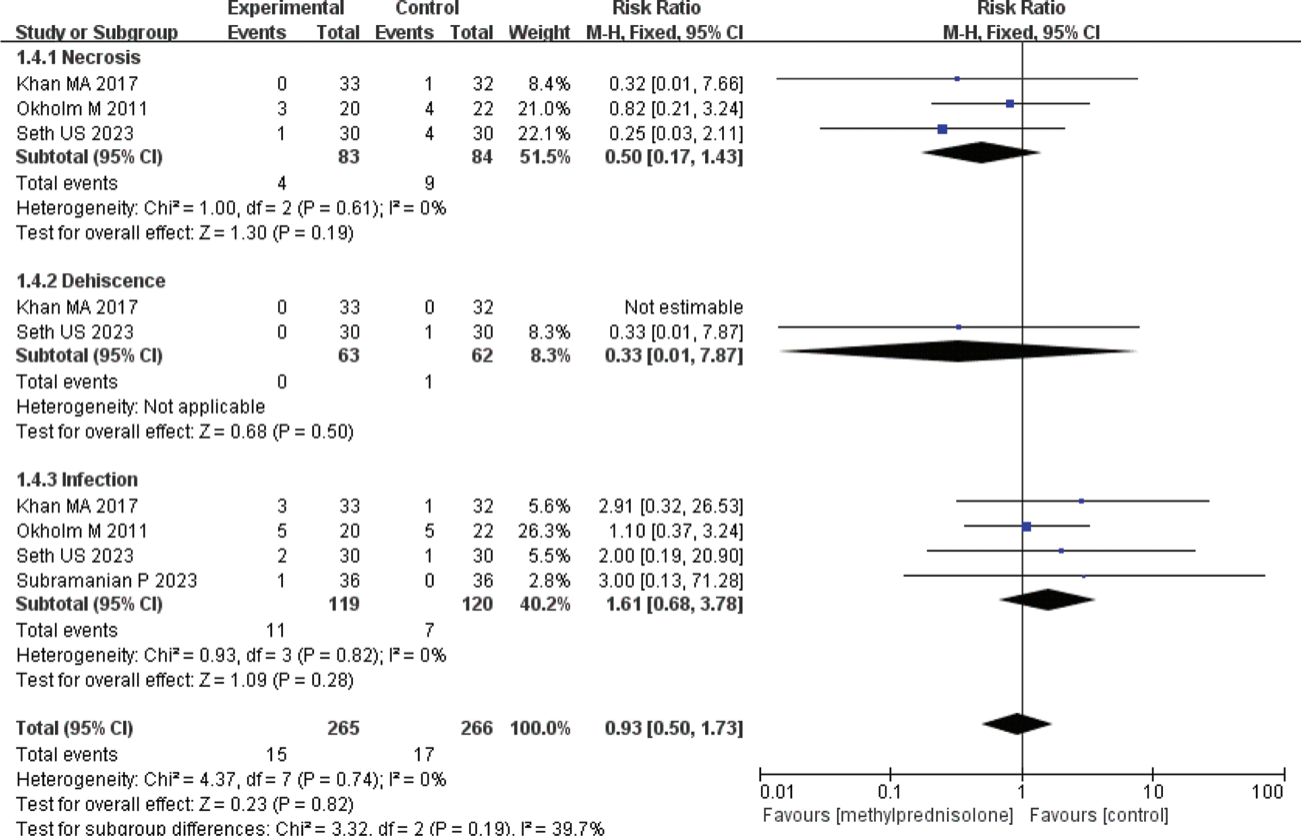
Forest plot of wound complications.

Four studies^[10,14,16,18]^ reported wound complications and there was no heterogeneity between the studies (*P* = .74, *I*^2^ = 0%) (Fig. 11). Using a fixed-effects model for meta-analysis, the results showed that there was no statistically significant difference in the incidence of wound complications between the methylprednisolone group and the control group (RR = 0.93, 95% CI 0.50–1.73, *P* = .82) (Fig. 11). Subgroup analyses of the different types of wound complications were performed, and meta-analysis showed no statistically significant differences between the incidence of the methylprednisolone group and the control group, for necrosis (RR = 0.50, 95% CI 0.17–1.43, *P* = .19), dehiscence (RR = 0.33, 95% CI 0.01–7.87, *P* = .50) and infection (RR = 1.61, 95% CI 0.68–3.78, *P* = .28) (Fig. 11). The TSA indicated that the cumulative z-curve did not cross the boundary for monitoring the study sequence or the boundary for futility, indicating that the current evidence was insufficient and inconclusive.

### 3.7. Seroma grading

Two studies^[14,16]^ reported seroma grading. There was no heterogeneity between studies for grade 1 (*P* = .61, *I*^2^ = 0%), grade 2 (*P* = .68, *I*^2^ = 0%) or grade 3 (*P* = .83, *I*^2^ = 0%) (Table 5). The results of the meta-analysis showed that there was no statistically significant difference in the incidence of grade 1 (RR = 0.49, 95% CI 0.13–1.88, *P* = .30), grade 2 (RR = 0.33, 95% CI 0.07–1.57, *P* = .16), or grade 3 (RR = 0.42, 95% CI 0.06–2.77, *P* = .37) seroma between the methylprednisolone group and the control group (Table 5). The TSA indicated that the cumulative z-curve did not cross the boundary for monitoring study progress or futility, indicating that the current evidence was insufficient and inconclusive.

**Table 5:** Meta-analysis results of seroma grading.

Grade	Number of included studies	Heterogeneity		Model	Statistical results		
		*P*	*I*^2^, %		RR	95% CI	*P*
Grade 1	2	.61	0	Fixed-effects model	0.49	0.13–1.88	.30
Grade 2	2	.68	0	Fixed-effects model	0.33	0.07–1.57	.16
Grade 3	2	.83	0	Fixed-effects model	0.42	0.06–2.77	.37

### 3.8. The GRADE evidence and summary of findings

The strength of the evidence for the main outcomes is shown in Table 6. Evidence quality was assessed as “moderate” for incidence of seroma, total drainage volume, duration of drainage, and wound complications, and “low” for seroma grading.

**Table 6:** The GRADE evidence and summary of findings.

Certainty assessment							№ of patients		Effect		Certainty
№ of studies	Study design	Risk of bias	Inconsistency	Indirectness	Imprecision	Other considerations	Methylprednisolone	Control	Relative (95% CI)	Absolute (95% CI)	
Incidence of seroma											
5	Randomized trials	Serious	Not serious	Not serious	Not serious	None	89/238 (37.4%)	130/237 (54.9%)	RR 0.68 (0.52–0.88)	176 fewer per 1000 (from 263 fewer to 66 fewer)	⨁⨁⨁◯ Moderate
Total drainage volume											
4	Randomized trials	Serious	Not serious	Not serious	Not serious	None	130	129	-	MD 184.19 lower (215.3 lower to 153.09 lower)	⨁⨁⨁◯ Moderate
Duration of drainage											
3	Randomized trials	Serious	Not serious	Not serious	Not serious	None	99	98	-	MD 3.37 lower (3.98 lower to 2.75 lower)	⨁⨁⨁◯ Moderate
Wound complications											
4	Randomized trials	Serious	Not serious	Not serious	Serious	None	15/265 (5.7%)	17/266 (6.4%)	RR 0.93 (0.50–1.73)	4 fewer per 1000 (from 32 fewer to 47 more)	⨁⨁◯◯ Low
Seroma grading: Grade 1											
2	Randomized trials	Serious	Not serious	Not serious	Serious	None	3/63 (4.8%)	6/62 (9.7%)	RR 0.49 (0.13–1.88)	49 fewer per 1000 (from 84 fewer to 85 more)	⨁⨁◯◯ Low
Seroma grading: Grade 2											
2	Randomized trials	Serious	Not serious	Not serious	Serious	None	2/63 (3.2%)	6/62 (9.7%)	RR 0.33 (0.07–1.57)	65 fewer per 1000 (from 90 fewer to 55 more)	⨁⨁◯◯ Low
Seroma grading: Grade 3											
2	Randomized trials	Serious	Not serious	Not serious	Serious	None	1/63 (1.6%)	3/62 (4.8%)	RR 0.42 (0.06–2.77)	28 fewer per 1000 (from 45 fewer to 86 more)	⨁⨁◯◯ Low

CI = confidence interval, MD = mean difference, RR = risk ratio.

## 4. Discussion

In this systematic review, we included 7 RCTs and primarily found that methylprednisolone may reduce the risk of seroma formation in patients undergoing mastectomy, as well as the total drainage volume and the duration of drainage. In addition, it may not affect the incidence of wound complications or seroma grading.

Despite significant advances in surgical practices, seroma formation following mastectomy for breast cancer remains a major and unavoidable problem.^[19]^ It is associated with limited movement of the ipsilateral hand and overall reduced mobility due to the presence of drains.^[20,21]^ Surgery induces a systemic inflammatory response, primarily characterized by the release of cytokines. In the following days, there is an increase in fibrinolytic activity and serum fluid.^[22–24]^ The pathogenesis of seroma is therefore thought to involve the accumulation of the exudative phase of the inflammatory response, characterized by inflammatory cells, immunoglobulins, and high levels of Interleukin 6 (IL-6).^[25,26]^

Steroids can be employed to suppress the inflammatory response, reduce seroma formation, and potentially improve the prognosis of patients following mastectomy. They achieve this by inhibiting the function of cytokines.^[27]^ One study found that local administration of 80 mg of methylprednisolone significantly diminishes seroma formation after abdominoplasty and reduces the level of IL-6 in the seroma fluid.^[28]^ The suppression of inflammatory markers is clinically equivalent to a reduction in seroma volume.^[28]^ In another study involving patients undergoing breast reconstruction, 55% of patients in the methylprednisolone injection group did not develop a seroma. In contrast, 95% of patients in the 0.9% normal saline injection group developed seroma.^[29]^

One of the side effects of steroids is the risk of infection and complicated wound healing. In an animal study, a local injection of 30 mg/kg of methylprednisolone sodium succinate was administered under the flap after mastectomy in a rat model. On the 7th day after mastectomy, axillary lymph node dissection was performed, and the volume of the seroma was recorded. This study concluded that although methylprednisolone is effective in preventing seroma, it should not be universally used due to the high risk of wound infection.^[9]^ However, our study found that there were no differences between the methylprednisolone group and the control group in terms of wound infection, necrosis, and dehiscence, which is consistent with other previous research findings.^[29,30]^

This study has some limitations: Although an extensive search was conducted across available databases, the number of clinical trials focusing on the use of methylprednisolone to prevent seroma after mastectomy was limited, and the sample size for certain indicators was thus small. TSA indicated that current evidence for the inclusion of patients with wound complications and seroma grading was insufficient and inconclusive. The intervention measures in some trials were not fully consistent, leading to clinical heterogeneity in medication regimens and follow-up times. Standardizing medication regimens and unified outcome measures are recommended in future research. Due to the limited sample size of the included trials, further large-scale randomized controlled trials are needed to validate our findings.

## 5. Conclusions

In conclusion, the findings of this study suggest that methylprednisolone may reduce the risk of seroma formation in patients undergoing mastectomy, along with reducing the total volume and duration of drainage. Further well-designed randomized controlled trials are needed to assess the effects of methylprednisolone on postoperative wound complications and seroma grading.

## Author contributions

**Conceptualization:** Xiangzun Xiong, Xiaoyuan Zheng.

**Data curation:** Xiangzun Xiong, Xue Ou, Lei Wang.

**Formal analysis:** Xiangzun Xiong, Xiaoyuan Zheng.

**Writing – original draft:** Xiangzun Xiong, Xue Ou, Lei Wang.

**Writing – review & editing:** Xiaoyuan Zheng.

## Supplementary Material


