# Supplementary material for: Efficacy and safety of methylprednisolone in the prevention of seroma formation after mastectomy: Systematic review and meta-analysis
Source: Medicine (Baltimore). 2025 Oct 24;104(43):e45353. doi: 10.1097/MD.0000000000045353 (PMC12558233; doi:10.1097/MD.0000000000045353)
Supplement: Supplementary file 1 [file medi-104-e45353-s001.doc]

Supplementary Table 1 Search strategy of all databases

| Database | Strategy |
| --- | --- |
| PubMed | (((((Steroids[Title/Abstract])) OR (Steroids[Mesh])) OR ((((((((Metipred[Title/Abstract])) OR (6-Methylprednisolone[Title/Abstract])) OR (“6 Methylprednisolone”[Title/Abstract])) OR (Urbason[Title/Abstract])) OR (Medrol[Title/Abstract])) OR (methylprednisolone[Title/Abstract])) OR (Methylprednisolone[Mesh]))) AND (((Seroma[Title/Abstract])) OR (Seroma[Mesh]))) AND ((((((Mastectomy[Title/Abstract])) OR (Mastectomies[Title/Abstract])) OR (Mammectomy[Title/Abstract])) OR (Mammectomies[Title/Abstract])) OR (Mastectomy[Mesh])) |
| Web of Science | (((TS=(mastectomy AND (methylprednisolone OR Steroids) AND Seroma)) OR TS=(mastectomy AND (methylprednisolone OR Steroids) AND Seroma)) OR TI=(mastectomy AND (methylprednisolone OR Steroids) AND Seroma)) OR AB=(mastectomy AND (methylprednisolone OR Steroids) AND Seroma) and Preprint Citation Index (Exclude – Database) |
| Embase | #1 'mastectomy'/exp  #2'amputation, breast':ab,ti OR 'breast amputation':ab,ti OR 'breast excision':ab,ti OR 'breast extirpation':ab,ti OR 'breast removal':ab,ti OR 'breast resection':ab,ti OR 'mammectomy':ab,ti OR 'resection, breast':ab,ti OR 'mastectomy':ab,ti  #3 'methylprednisolone'/exp  #4 '11, 17 dihydroxy 17':ab,ti AND '2 hydroxyacetyl':ab,ti AND '6, 10, 13 trimethyl 7, 8, 9, 11, 12, 14, 15, 16 octahydro 6h cyclopenta [a] phenanthren 3 one':ab,ti OR '11, 17, 21 trihydroxy 6 methylpregna 1, 4 diene 3, 20 dione':ab,ti OR '11beta, 17alpha, 21 trihydroxy 6alpha methyl 1, 4 pregnadiene 3, 20 dione':ab,ti OR '2 methylprednisolone':ab,ti OR '5 methylprednisolone':ab,ti OR '6 alpha methylprednisolone':ab,ti OR '6 methyl delta 1 hydrocortisone':ab,ti OR '6 methyl prednisolone':ab,ti OR '6 methylprednisolone':ab,ti OR '6alpha methyl 11beta, 17alpha, 21 triol 1, 4 pregnadiene 3, 20 dione':ab,ti OR '6alpha methyl delta1 hydrocortisone':ab,ti OR '6alpha methylprednisolone':ab,ti OR 'adlone-40':ab,ti OR 'adlone-80':ab,ti OR 'beta methylprednisolone':ab,ti OR 'dep medalone 80':ab,ti OR 'depmedalone':ab,ti OR 'depoject-80':ab,ti OR 'depopred':ab,ti OR 'esametone':ab,ti OR 'firmacort':ab,ti OR 'med-jec-40':ab,ti OR 'medixon':ab,ti OR 'mednin':ab,ti OR 'medralone 80':ab,ti OR 'medrate':ab,ti OR 'medrol':ab,ti OR 'medrol a':ab,ti OR 'medrol adt pak':ab,ti OR 'medrol compositum':ab,ti OR 'medrol dosepak':ab,ti OR 'medrol medules':ab,ti OR 'medrol pak':ab,ti OR 'medrone':ab,ti OR 'meprednisolone':ab,ti OR 'meprelon':ab,ti OR 'mesopren':ab,ti OR 'methacort 40':ab,ti OR 'methacort 80':ab,ti OR 'methyl prednisolone':ab,ti OR 'methylcotol':ab,ti OR 'methylcotolone':ab,ti OR 'methylpred dp':ab,ti OR 'methylsterolone':ab,ti OR 'metidrol':ab,ti OR 'metrisone':ab,ti OR 'metycortin':ab,ti OR 'metypred':ab,ti OR 'metypresol':ab,ti OR 'neomedrone':ab,ti OR 'nsc 19987':ab,ti OR 'nsc19987':ab,ti OR 'prednol':ab,ti OR 'solomet':ab,ti OR 'solu decortin':ab,ti OR 'urbason':ab,ti OR 'methylprednisolone':ab,ti  #5 'steroid'/exp  #6 'cyclosteroids':ab,ti OR 'steroid compound':ab,ti OR 'steroid derivative':ab,ti OR 'steroidal compound':ab,ti OR 'steroids':ab,ti OR 'steroids, brominated':ab,ti OR 'steroids, chlorinated':ab,ti OR 'steroid':ab,ti  #7 'seroma'/exp  #8 'seroma':ab,ti  #9 #1 OR #2  #10 #3 OR #4 OR #5 OR #6  #11 #7 OR #8  #12 #9 AND #10 AND #11 |
| Cochrane Library | #1 MeSH descriptor. [Mastectomy] explode all trees  #2 (Mammectomies).ti,ab,kw OR (Mammectomy):ti,ab,kw OR (Mastectomies):ti,ab,kw OR (mastectomy):ti.ab,kw  #3 MeSH descriptor: [Methylprednisolone] explode all trees  #4 (Urbason):ti,ab,kw OR (Metipred):ti,ab,kw OR (methylprednisolone):ti,ab,kw OR (Medrol):ti.ab.kw  #5 MeSH descriptor: [Steroids] explode all trees  #6 (Steroid):ti,ab,kw OR (Steroids):ti,ab,kw OR (catatoxic):ti,ab,kw  #7 MeSH descriptor: [Seroma] explode all trees  #8 (Seroma):ti,ab,kw OR (Seromas):ti,ab,kw  #9 #1 OR #2  #10 #3 OR #4 OR #5 OR #6  #11 #7 OR #8  #12 #9 AND #10 AND #11 |
| CNKI | (SU=(血清肿) AND (甲强龙 OR 甲泼尼龙 OR 类固醇 OR 糖皮质激素)) OR (TI=(血清肿) AND ( 甲强龙 OR 甲泼尼龙 OR 类固醇 OR 糖皮质激素)) OR (KY=(血清肿) AND ( 甲强龙 OR 甲泼尼龙 OR 类固醇 OR 糖皮质激素)) OR (AB=(血清肿) AND ( 甲强龙 OR 甲泼尼龙 OR 类固醇 OR 糖皮质激素)) |
| Wanfang Data | ((题名=血清肿) AND 题名=(甲强龙 OR 甲泼尼龙 OR 类固醇 OR 糖皮质激素)) OR ((关键词=血清肿) AND 关键词=(甲强龙 OR 甲泼尼龙 OR 类固醇 OR 糖皮质激素)) OR ((摘要=血清肿) AND摘要=(甲强龙 OR 甲泼尼龙)) OR ((主题=血清肿) AND 主题=(甲强龙 OR 甲泼尼龙 OR 类固醇 OR 糖皮质激素)) |
| CBM | "血清肿"[常用字段:智能] AND( "甲强龙"[常用字段:智能] OR "甲泼尼龙"[常用字段:智能] OR "类固醇"[常用字段:智能] OR "糖皮质激素"[常用字段:智能]) |
| VIP | (R=(血清肿) AND (甲强龙 OR 甲泼尼龙 OR 类固醇 OR 糖皮质激素)) OR (M=(血清肿) AND (甲强龙 OR 甲泼尼龙 OR 类固醇 OR 糖皮质激素)) |
